# A conserved leucine zipper-like motif accounts for strong tetramerization capabilities of SEPALLATA-like MADS-domain transcription factors

**DOI:** 10.1093/jxb/ery063

**Published:** 2018-02-21

**Authors:** Florian Rümpler, Günter Theißen, Rainer Melzer

**Affiliations:** 1 Department of Genetics, Friedrich Schiller University Jena, Philosophenweg, Jena, Germany; 2 School of Biology and Environmental Science, University College Dublin, Belfield, Dublin, Irel

**Keywords:** *Arabidopsis thaliana*, coiled-coil, floral quartet, flower development, keratin-like domain, MADS-box gene, MIKC-type protein, SEPALLATA3

## Abstract

The development of angiosperm flowers is regulated by homeotic MIKC-type MADS-domain transcription factors that activate or repress target genes via the formation of DNA-bound, organ-specific tetrameric complexes. The protein–protein interaction (PPI) capabilities differ considerably between different MIKC-type proteins. In *Arabidopsis thaliana* the floral homeotic protein SEPALLATA3 (SEP3) acts as a hub that incorporates numerous other MADS-domain proteins into tetrameric complexes that would otherwise not form. However, the molecular mechanisms that underlie these promiscuous interactions remain largely unknown. In this study, we created a collection of amino acid substitution mutants of SEP3 to quantify the contribution of individual residues on protein tetramerization during DNA-binding, employing methods of molecular biophysics. We show that leucine residues at certain key positions form a leucine-zipper structure that is essential for tetramerization of SEP3, whereas the introduction of physicochemically very similar residues at respective sites impedes the formation of DNA-bound tetramers. Comprehensive molecular evolutionary analyses of MADS-domain proteins from a diverse set of flowering plants revealed exceedingly high conservation of the identified leucine residues within SEP3-subfamily proteins throughout angiosperm evolution. In contrast, MADS-domain proteins that are unable to tetramerize among themselves exhibit preferences for other amino acids at homologous sites. Our findings indicate that the subfamily-specific conservation of amino acid residues at just a few key positions accounts for subfamily-specific interaction capabilities of MADS-domain transcription factors and this has shaped the present-day structure of the PPI network controlling flower development.

## Teaser

The strong tetramerization capabilities of the floral homeotic MADS-domain protein SEP3 largely depend on leucine residues at just a few key positions within the protein–protein interacting keratin-like domain.

## Introduction

Complexity of biological systems is often achieved by the combined activity of a small number of factors (Reményi *et al.*, 2004). One important example is represented by protein–protein interaction (PPI) networks that are based on transcription factors (TFs) that act in a combinatorial manner to accomplish the required degree of (e.g.) morphological complexity. PPI networks often approximate a scale-free structure (Barabási and Oltvai, 2004). They contain a small number of hub proteins with many interaction partners and a large number of poorly connected nodes. Although combinatorial control is of eminent importance for almost all developmental processes, the molecular determinants that underlie the specific combinatorial interactions remain poorly understood. This is especially true for PPIs among transcription factors (TFs) belonging to the same family. The respective TFs are often very similar in terms of sequence and biochemical properties, yet they fulfill highly distinct and specific functions that are at least partially determined by distinct protein–protein interactions. The PPI network controlling flower development in angiosperms is a good case in point. Floral organ specification is regulated by so-called floral quartets, which are organ-specific tetrameric complexes of MIKC-type MADS-domain TFs bound to two adjacent DNA-binding sites while looping the DNA to regulate target genes (Melzer and Theissen, 2009; Melzer *et al.*, 2009; Theißen *et al.*, 2016; Theissen and Saedler, 2001). In the model plant species *Arabidopsis thaliana* the floral homeotic protein SEPALLATA3 (SEP3) together with its paralogs SEP1, SEP2, and SEP4 from the closely related LOFSEP-subfamily bears a central role by forming tetrameric complexes with numerous other MIKC-type MADS-domain TFs (Immink *et al.*, 2009; Melzer and Theissen, 2009; Smaczniak *et al.*, 2012; Zahn *et al.*, 2005). The four SEP proteins act in a largely redundant manner but, in agreement with their central position in the PPI network controlling flower development, *sep* multiple-mutants show severe developmental defects (Pelaz *et al.*, 2000; Ditta *et al.*, 2004). *sep1 sep2 sep3* triple-mutant plants develop sepals from primordia that would normally develop into petals, stamens, and carpels, and *sep1 sep2 sep3 sep4* quadruple-mutants develop vegetative leaves instead of floral organs (Pelaz *et al.*, 2000; Ditta *et al.*, 2004).

Among the four *SEP* genes, *SEP3* has been the best studied (Favaro *et al.*, 2003; Immink *et al.*, 2009; Kaufmann *et al.*, 2009; Melzer and Theissen, 2009; Melzer *et al.*, 2009; Smaczniak *et al.*, 2012; Jetha *et al.*, 2014; Muiño *et al.*, 2016; Gusewski *et al.*, 2017). Beyond the formation of complexes that determine floral organ identity, SEP3 is also involved in controlling flowering time, floral transition, and ovule development (Favaro *et al.*, 2003; Immink *et al.*, 2009; Liu *et al.*, 2009; Lopez-Vernaza *et al.*, 2012). It therefore constitutes one of the major hub proteins within the PPI network that controls reproductive development (Favaro *et al.*, 2003; Immink *et al.*, 2009; Liu *et al.*, 2009; Smaczniak *et al.*, 2012). However, it is unclear which biochemical and biophysical properties enable SEP3 to form DNA-bound tetramers with numerous partners whereas other MIKC-type MADS-domain TFs are unable to form floral quartet-like complexes among themselves. For example, the floral homeotic proteins APETALA3 (AP3) and PISTILLATA (PI) from *A. thaliana* that are involved in the developmental specification of petals and stamens only form obligate heterodimers and require SEP proteins for tetramer formation (Immink *et al.*, 2009; Melzer and Theissen, 2009; Melzer *et al.*, 2014).

**Fig. 1.**
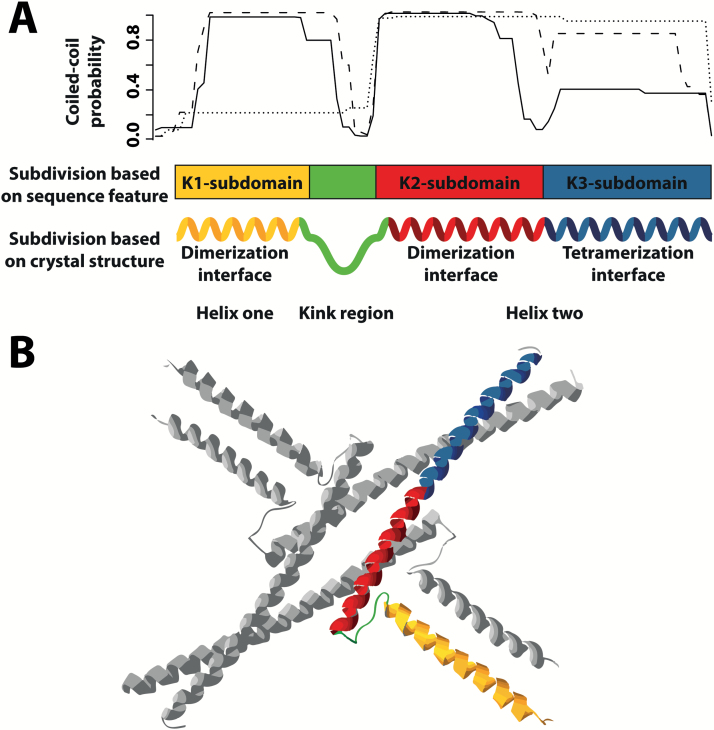
Architecture of the K-domain of SEP3 based on sequence and structural features. (A) Based on coiled-coil predictions (top), the K-domain was assumed to fold into three separate coiled-coils and was thus subdivided into three subdomains: K1, K2, and K3 (middle). The crystal structure of the K-domain of SEP3 revealed that it folds into two α-helices separated by a kink region (bottom). The first helix spans the K1-subdomain (color-coded in yellow) and is involved in the dimerization of two SEP3 monomers (i.e. dimerization interface). The second helix spans the K2- and K3-subdomains and constitutes an N-terminal interaction interface that further stabilizes dimerization of two SEP3 monomers (red) and a second C-terminal interaction interface that mediates the interaction of two SEP3 dimers (i.e. tetramerization interface, blue). Coiled-coil predictions were performed with COILS (Lupas *et al.*, 1991). The solid, dashed and dotted lines in the graph at the top correspond to a sliding window size of 14, 21, and 28 amino acids used for the prediction, respectively. (B) Crystal structure of a SEP3 K-domain homotetramer (PDB ID: 4ox0, https://www.rcsb.org/; Puranik *et al.*, 2014). The dimerization interface of helix one, the kink region, the dimerization interface of helix two, and the tetramerization interface of one K-domain are color-coded in yellow, green, red, and blue, respectively.

The PPIs that allow for tetramer formation are mainly mediated by the ~80 amino acid-long keratin-like domain (K-domain), which is shared by all MIKC-type MADS-domain TFs (Yang *et al.*, 2003; Yang and Jack, 2004; Melzer and Theissen, 2009). The amino acid sequence within the K-domain of most MADS-domain proteins shows three characteristic heptad-repeat patterns (K1-; K2-; K3-subdomain repeat) of the form [abcdefg]_*n*_, where most ‘a’ and ‘d’ positions are occupied by highly hydrophobic residues (Riechmann and Meyerowitz, 1997; Yang *et al.*, 2003; Yang and Jack, 2004). This sequence feature is typical for coiled-coils, a common and intensively studied type of PPI domain (Betz *et al.*, 1995; Mason and Arndt, 2004; Parry *et al.*, 2008; Mason *et al.*, 2009) (Fig. 1). Within a coiled-coil, an α-helix is formed and the amino acids on the heptad-repeat ‘a’ and ‘d’ positions form a stripe of hydrophobic residues that runs along the α-helix and facilitates hydrophobic interaction with a partner coiled-coil (Mason and Arndt, 2004; Mason *et al.*, 2009).

Recently, the crystal structure of the complete K-domain of SEP3 was reported (Puranik *et al.*, 2014). Based on the crystal structure, the K-domain forms two amphipathic α-helices separated by a kink region that prevents intramolecular association of both helices. Helix one comprises the first heptad-repeat (K1-subdomain) and is involved in dimerization of two SEP3 monomers. Helix two spans heptad-repeat two (K2-subdomain) that further stabilizes the interaction of two SEP3 monomers and heptad-repeat three (K3-subdomain), which constitutes an interface for the interaction of two SEP3 dimers, i.e. tetramerization (Fig. 1).

In this study, we determined the sequence features that enable SEP3 to form tetrameric complexes and identified the amino acid patterns that distinguish members of the SEP3 subfamily from other MIKC-type MADS-domain TFs with more restricted tetramerization capabilities. Our data suggest that leucine residues at intramolecular contact points and at the interaction interface of the K3-subdomain are indispensable for tetrameric complex formation. Sequence analyses of MIKC-type MADS-domain TFs from a broad set of flowering plant species revealed very high conservation of the examined leucine residues in the SEP3 subfamily throughout angiosperm evolution. In contrast, members of other MIKC-type MADS-domain TF subfamilies such as AP3 and PI showed preferences for other amino acids at homologous sites. The identified leucines may thus be a critical denominator that determines the ability of SEP3-subfamily proteins to incorporate a great number of other MIKC-type MADS-TFs into floral quartets.

## Methods

### Cloning procedures and site-directed mutagenesis

The plasmids for *in vitro* transcription/translation of *A. thaliana SEP3* (NCBI accession NM_102272), *AP3* (NM_115294), *PI* (NM_122031), and *Amborella trichopoda AMtrAGL9* (KF925502), namely pTNT-SEP3, pSPUTK-AP3, pSPUTK-PI, and pSPUTK-AMtrAGL9, were generated previously (Melzer *et al.*, 2009, 2014). The cDNA sequences for the single- and double-amino acid substitution mutants of SEP3 were created by site-directed mutagenesis PCR using the Q5 Site-Directed Mutagenesis Kit (New England Biolabs) according to the manufacturer’s instructions. The cDNA sequence for the chimeric protein SEP3_AP3chim_ was created by megaprimer-mediated mutagenesis PCR for domain substitutions according to Perez *et al.* (2006).

### Design of DNA probes and radioactive labeling

Design and preparation of DNA probes have been described previously (Melzer *et al.*, 2009). The CArG-box sequence 5′-CCAAATAAGG-3′ that was used for all DNA probes was derived from the regulatory intron of *AGAMOUS*. For studies on homotetramer formation, a 151-nt DNA probe was used that contained two CArG-boxes within a distance of 63 bp, i.e. six helical turns (sequence: 5′-TCGAG GTCGG AAATT TAATT ATATT *CCAAA TAAGG* AAAGT ATGGA ACGTT CGACG GTATC GATAA GCTTG ATGAA ATTTA ATTAT ATT*CC AAATA AGG*AA AGTAT GGAAC GTTAT CGAAT TCCTG CAGCC CGGGG GATCC ACTAG TTCTA G-3′; CArG-box sequences are in italics). Saturation binding assays to quantify dimer binding affinities were performed with a 51-nt DNA probe harboring a single CArG-box in the center (sequence: 3′-AATTC GAAAT TTAAT TATAT T*CCAA ATAAG G*AAAG TATGG AACGT TGAAT T-5′; CArG-box sequence is in italic). The DNA probes were radioactively labeled via a Klenow fill-in reaction of 5′-overhangs with [α-^32^P] dATP.

### *In vitro* transcription/translation and electrophoretic mobility shift assay

Proteins were produced *in vitro* using the TNT SP6 Quick Coupled Transcription/Translation System (Promega) according to the manufacturer’s instructions, and used directly without freezing and thawing. The composition of the protein–DNA binding reaction buffer was essentially as described by Egea-Cortines *et al.* (1999), with final concentrations of 1.6 mM EDTA, 10.3 mM HEPES, 1 mM DTT, 1.3 mM Spermidine hydrochloride, 33.3 ng µl^–1^ Poly dI/dC, 2.5 % CHAPS, 4.3 % glycerol, and a minimum of 1.3 µg µl^–1^ BSA. The amounts of protein, DNA probe, and BSA were varied according to the assay being performed. For co-operative DNA-binding studies to infer tetramer formation capabilities, a constant amount of 0.1 ng of a DNA probe containing two CArG-boxes within a distance of six helical turns was co-incubated with variable amounts of *in vitro* translated protein, ranging from 0.05 µl to 3 µl. Variable amounts of applied *in vitro* translated protein were compensated by adding appropriate volumes of BSA (10 µg µl^–1^). For saturation binding assays to quantify dimer binding affinities, a constant amount of 2–5 µl *in vitro* translated protein was co-incubated with variable amounts of a DNA probe containing one CArG-box in the center, ranging from 0.05–32 ng as previously described in Jetha *et al.* (2014). Binding reactions had a total volume of 12 µl, and were incubated overnight at 4 °C and subsequently loaded on a polyacrylamide (5 % acrylamide, 0.1725 % bisacrylamid) 0.5× TBE gel that had been pre-run for 30 min. The gel was run at room temperature with 0.5× TBE buffer for 2.5 h at 7.5 V cm^–1^, and afterwards dried and exposed onto a phosphorimaging screen to quantify signal intensities.

### Quantification of co-operative DNA-binding

For each lane of the EMSA gel, relative signal intensities of all fractions were measured using Multi Gauge 3.1 (Fujifilm). The equations that were used to quantify the ability for co-operative DNA-binding of two dimers to a DNA probe carrying two CArG-boxes have been described previously (Melzer *et al.*, 2009; Senear and Brenowitz, 1991). Briefly, if the relative concentration of unbound DNA probe [*Y*_0_] (signal of high electrophoretic mobility), a DNA probe bound by two proteins [*Y*_2_] (signal of intermediate electrophoretic mobility), and a DNA probe bound by four proteins [*Y*_4_] (signal of low electrophoretic mobility) are described as a function of applied protein [P2]:

$$\left[Y_{0}\right]=\;\frac{1}{1+\left(\frac{2}{k_{\text{d}1}}\right)\times \left[\text{P}2\right]+\left(\frac{1}{k_{\text{d}1}\times k_{\text{d}2}}\right)\times {\left[\text{P}2\right]}^{2}}$$ (1)

$$\left[Y_{2}\right]=\;\frac{\left(\frac{2}{k_{d1}}\right)\times \left[\text{P}2\right]}{1+\left(\frac{2}{k_{\text{d}1}}\right)\times \left[\text{P}2\right]+\left(\frac{1}{k_{\text{d}1}\times k_{\text{d}2}}\right)\times {\left[\text{P}2\right]}^{2}}$$ (2)

$$\left[Y_{4}\right]=\;\frac{\left(\frac{1}{k_{\text{d}1}\times k_{\text{d}2}}\right)\times {\left[\text{P}2\right]}^{2}}{1+\left(\frac{2}{k_{\text{d}1}}\right)\times \left[\text{P}2\right]+\left(\frac{1}{k_{\text{d}1}\times k_{\text{d}2}}\right)\times {\left[\text{P}2\right]}^{2}}$$ (3)

then *k*_d1_ is the dissociation constant for binding of a protein dimer to a DNA probe with two unoccupied binding sites, and *k*_d2_ is the dissociation constant for binding of a second protein dimer to a DNA probe where one of the two binding sites is already occupied. By non-linear regression of the measured signal intensities of the three fractions to equations (1) to (3), *k*_d1_ and *k*_d2_ were estimated using GraphPad Prism 5 (GraphPad Software). As we used *in vitro* transcription/translation for protein production, the exact protein concentrations were unknown. Therefore, the amount of applied *in vitro* transcription/translation mixture was used as proxy for [P2], as previously described (Melzer *et al.*, 2009). As a result of the unknown protein concentrations, the estimated values for *k*_d1_ and *k*_d2_ depend on the *in vitro* transcription/translation efficiency and can only be considered as relative values. However, estimating a co-operativity constant *k*_coop_ (defined as the ratio of *k*_*d1*_ and *k*_*d2*_) is still possible:

$$k_{\text{coop}}=\;\frac{k_{\text{d}1}}{k_{\text{d}2}}$$ (4)

As discussed elsewhere (Senear *et al.*, 1993; Jetha *et al.*, 2014), the determination of *k*_coop_ values critically depends on the detection of single dimers bound to DNA, leading to some variation in the determination of especially high *k*_coop_ values. As described previously, *k*_coop_ values of ≈200 were the upper limit that could be determined with our experimental setup (Jetha *et al.*, 2014).

### Saturation binding assay

To estimate the dissociation constant for binding of a protein dimer to a single DNA-binding site, *k*_d_, saturation binding assays with a DNA probe carrying a single CArG-box were performed. The equation that was used to infer *k*_d_ has been described previously (Jetha *et al.*, 2014). *k*_d_ can be defined as:

$$k_{\text{d}}=\;\frac{\left(\left[{\text{P}}_{\text{t}}\right]-[\text{PD}]\right)\times [\text{D}]}{\left[\text{PD}\right]}$$ (5)

with [PD], [P_t_], and [D] being the concentration of the protein–DNA complex, total protein, and unbound DNA probe, respectively. By expressing [PD] as a function of [D] for increasing concentrations of applied DNA probe, [P_t_] and *k*_d_ were determined via non-linear regression using GraphPad Prism 5.

### Multiple sequence alignments and *in silico* sequence analysis

For analyses of amino acid preferences of different MIKC-type MADS-domain protein subfamilies throughout the K-domain, a comprehensive sequence collection was compiled. Via BLAST searches (Altschul *et al.*, 1990), representatives of all 14 subfamilies (Becker and Theissen, 2003; Gramzow and Theißen, 2013) of MIKC-type proteins present in *A. thaliana* (AP1-, AP3-, PI-, AG-, ABS-, SEP3-, LOFSEP-, AGL6-, AGL12-, AGL15-, AGL17-, FLC-, SOC1-, and SVP-subfamily) were collected using the amino acid sequences of *A. thaliana* AP1, AP3, PI, AG, ABS, SEP3, SEP1, AGL6, AGL12, AGL15, AGL17, FLC, SOC1, and SVP, respectively, as queries. To cover a broad set of species, six individual searches were performed for each subfamily. Each of those searches was restricted to a different group of seed plants: core eudicots, early-diverging eudicots, monocots, magnoliids, early-diverging angiosperms, and gymnosperms. For sequences from core eudicots, the search queries were restricted to asterids (BLAST tax-ID: 71274), Dilleniaceae (24942), Caryophyllidea (108240), Santalales (41947), Berberidopsidales (403664), Saxifragales (41946), rosids (71275), and Gunnerales (232382); for sequences from early-diverging eudicots, the search queries were restricted to Proteales (232378), Buxales (280577), and Ranunculales (41768); for sequences from monocots and magnoliids, respectively, the queries were restricted to the corresponding pre-defined organism groups implemented in BLAST (tax-ID: 4447 and 232347, respectively); for sequences from early-diverging angiosperms, the queries were restricted to Austrobaileyales (82956), Hydatellaceae (178426), Nymphaeales (261007), and Amborella (13332); and for sequences from gymnosperms, the queries were restricted to Gnetales (3378), Pinaceae (3318), Taxaceae (25623), Cephalotaxus (50178), Cupressaceae (3367), Araucariaceae (25664), Podocarpaceae (3362), Ginkgoales (3308), and Cycadales (3297). For each of the 84 resulting BLAST searches the amino acid sequences of all hits were downloaded (if more than 100 sequences were found, only the top 100 hits according to the total score calculated by BLAST were downloaded). The results of all BLAST searches were combined into a single data set, then all completely redundant sequences as well as all sequences that did not constitute MIKC-type proteins were removed and the remaining sequences were aligned with Mafft applying the E-INS-i mode using Jalview (Waterhouse *et al.*, 2009; Katoh and Standley, 2013). The subfamily assignment of each sequence was performed according to its clustering within a phylogenetic tree calculated with MrBayes (based on MADS-, I-, and K-domain sequences, applying a mixed AA model with 20 million generations, 50% burn-in, and a sample frequency of 1000) (Huelsenbeck and Ronquist, 2001). All sequences with uncertain subfamily assignment were removed. To optimize the alignment quality of the K-domain, 133 further sequences were removed that produced gaps and that did not appear to be representative for the respective subfamily. The final sequence collection comprised 1325 MIKC-type protein sequences.

Relative sequence similarities at homologous sites were calculated with R (www.R-project.org/). Each pair of amino acids at equivalent sites was assigned a similarity score based on BLOSUM40 values that were normalized to 1 by dividing by the maximum value of the respective amino acid. Subsequently, all pairwise similarity scores were averaged to calculate the mean relative sequence similarity for all amino acid positions within the K-domain. BLOSUM40 was chosen because the average sequence identity within the K-domain of all examined sequences was about 40 %. Box-plots and line graphs of sequence similarity scores were created with SPSS (IBM). The statistical significance of sequence similarity differences was tested using Mann–Whitney *U*-tests implemented in SPSS.

Subfamily-specific amino acid frequencies and mean hydrophobicity values for positions within the K-domain were calculated with R. SEP3 K-domain crystal structure pictures were created with Swiss-PdbViewer (SIB). Helical wheel diagrams were created with R. Coiled-coil predictions to pre-select potential positions for single- and double-amino acid substitutions were performed with COILS (Lupas *et al.*, 1991).

## Results

### Leucine residues in the K-domain strongly influence co-operative DNA-binding of SEP3

**Fig. 2.**
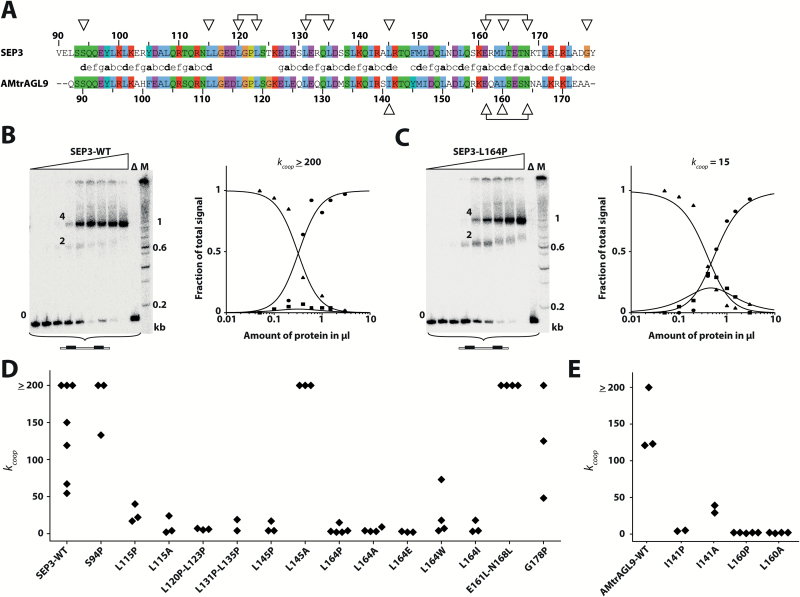
Ability of SEP3 and AMtrAGL9 wild-type proteins and different amino acid-substitution mutants to co-operatively bind to DNA. (A) Pairwise sequence alignment of the K-domains of SEP3 and AMtrAGL9; the heptad-repeat pattern is depicted between the two. The positions at which amino acids were substituted are indicated by triangles. (B, C) Binding of SEP3 wild-type (B) and SEP3-L164P (C) to a DNA probe containing two CArG-boxes. Increasing amounts of *in vitro* translated protein were incubated with constant amounts of DNA probe. As a negative control, the empty pTNT vector without any cDNA insert was used as template DNA for the *in vitro* translation (lane ∆). For size comparison, a radioactively labeled DNA ladder (100 bp DNA Ladder, New England BioLabs) was applied (lane M). The labeling of the three different fractions ‘0’, ‘2’, and ‘4’ corresponds to the number of proteins bound to one DNA molecule. Quantified signal intensities of the different fractions and graphs fitted according to equations (1) to (3) (see Methods) are shown next to the gel pictures (triangles, free DNA; squares, DNA probe bound by two proteins; circles, DNA probe bound by four proteins). The *k*_coop_ value inferred from these particular measurements are given above the graphs. (D, E) *k*_coop_ values for the wild-type protein and all examined single and double amino acid-substitution mutants of SEP3 (D) and AMtrAGL9 (E). *k*_coop_ values above 200 could not be determined reliably (see Methods).

To investigate the relevance of the different K-subdomains for co-operative DNA-binding and tetramer formation, single- and double-amino acid substitutions to proline were performed. Proline was chosen because it possesses helix-breaking properties (Richardson, 1981; Nilsson *et al.*, 1998) and would thus be expected to disrupt the overall structure of the respective K-subdomain. For each of the three K-subdomains, two substitution mutants were created (Fig. 2A, Supplementary Fig. S1 at JXB online). Based on coiled-coil predictions, one substitution mutant was supposed to destroy the K-subdomain coiled-coil (L115P for K1-, L131P-L135P for K2-, and L164P for K3-subdomain, respectively) whereas the other one was expected not to alter the formation of the respective coiled-coil [S94P (K1); L145P (K2); G178P (K3)]. Beyond the three K-subdomains, we also introduced proline substitutions at positions occupied by two conserved hydrophobic amino acids in the interhelical region between the K1- and the K2-subdomain (L120P and L123P, Fig. 2A) because L120 and L123 are homologous to L121 and V124 in the MADS-domain protein PI and those positions have been shown to be important for protein–protein interactions (Yang and Jack, 2004).

We used electrophoretic mobility shift assays (EMSAs) to study the DNA-binding and tetramerization behavior of the mutant SEP3 proteins. Based on previous studies it is known that SEP3 binds as a homodimer to a DNA-element termed CArG-box [for CCArichGG; consensus sequence 5′-CC(A/T)_6_GG-3′] and that four SEP3 proteins bind to a DNA probe containing two CArG-boxes (Melzer *et al.*, 2009). To first investigate whether the DNA-binding affinities of individual dimers were affected by the different amino acid substitutions, we performed saturation-binding EMSA experiments using increasing amounts of a DNA probe containing only one CArG-box together with constant amounts of protein, as previously described (Jetha *et al.*, 2014). The estimated affinities for binding of the altered SEP3 proteins to a single DNA-binding site varied slightly but did not considerably differ from the values obtained for SEP3 wild-type protein (Supplementary Fig. S2, Supplementary Table S1), indicating that the different amino acid substitutions did not, or only marginally, affect DNA-binding of individual dimers.

If increasing amounts of SEP3 were incubated together with constant amounts of a DNA probe containing two CArG-boxes, three bands of different electrophoretic mobility were observed (Fig. 2B, left panel). As determined previously (Melzer *et al.*, 2009) the band of high electrophoretic mobility constitutes unbound DNA (indicated with ‘0’ in Fig. 2B), the band of intermediate electrophoretic mobility constitutes a DNA probe bound by two SEP3 proteins (‘2’), and the band of low electrophoretic mobility constitutes a DNA probe bound by four SEP3 proteins (‘4’). By analysing the signal intensities of the three different fractions, the ability of SEP3 to form DNA-bound tetrameric complexes can be quantified and expressed via the co-operativity constant *k*_coop_ (equation 4 in Methods). *k*_coop_ equals 1 for non-cooperative binding and increases with increasing tetramer formation capabilities of the examined protein (e.g. a *k*_coop_ value of 100 indicates that the dissociation constant for the DNA-binding affinity of the second dimer is 100 times lower than the dissociation constant for the DNA-binding of the first dimer). SEP3 wild-type protein always showed a highly co-operative DNA-binding, producing *k*_coop_ values from 54 up to 200. The degree of co-operativity varied between different experiments and was slightly higher than previous estimates (Melzer *et al.*, 2009; Jetha *et al.*, 2014), probably owing to difficulties in precisely determining high *k*_coop_ values (Fig. 2B, D, Supplementary Table S1). As noted earlier, *k*_coop_ values of ≈200 were the upper limit that could be determined with our experimental set-up (Jetha *et al.*, 2014).

In contrast to the wild-type protein, all of the leucine-to-proline substitution mutants of SEP3 (L115P; L120P-L123P; L131P-L135P; L145P; L164P) showed a considerably reduced ability to bind co-operatively to DNA *in vitro*, independent of whether the formation of coiled-coils was predicted to be affected or not (Fig. 2C, D, Supplementary Table S1). Only the two proline substitutions S94P and G178P, located at the N- and C-terminal borders of the K-domain, respectively, did not strongly reduce co-operative binding of SEP3.

To test the effect of amino acid substitutions that are supposed to have a less severe effect on helix formation than proline, we substituted a subset of the previously selected leucines (L115; L145; L164) by alanine. Of these three substitutions, only L145A showed a co-operative DNA-binding ability comparable to that of SEP3 wild-type protein, whereas substitutions L115A and L164A caused an almost complete loss of co-operative DNA-binding, comparable to the proline substitutions at the respective positions (Fig. 2D, Supplementary Table S1). We further substituted position L164 by three additional amino acids (L164E; L164W; L164I) comprising glutamate and tryptophan, which occur at position 164 in several members of the SEP subfamily, and isoleucine, which has very similar physicochemical properties to leucine. However, none of the resulting mutants was able to approach the cooperative binding strength of the SEP3 wild-type protein (Fig. 2D, Supplementary Table S1). Our results indicate that the examined leucine residues are of critical importance for tetramer formation and co-operative binding of SEP3.

Within the [abcdefg]_*n*_ heptad-repeat of the K3-subdomain of SEP3, two neighboring ‘a’ positions (E161; N168) are not occupied by hydrophobic amino acids. Substituting these positions by leucine (E161L-N168L) resulted in a higher probability for the formation of the K3-subdomain coiled-coil *in silico* (see Supplementary Fig. S1). The respective mutant protein showed a co-operativity at least as high as the wild-type protein in EMSAs. In contrast to the wild-type protein, repeated measurements revealed that co-operativity was consistently above 200 (Fig. 2D, Supplementary Table S1). In fact, in none of the EMSAs we performed was a signal of a DNA probe bound by only one protein dimer detected, an observation that was different from the other proteins for which high co-operativity in DNA-binding was detected (e.g. SEP3-WT and SEP3-L145A), indicating that co-operative binding was increased by the E161L-N168L substitutions (Supplementary Fig. S3). Surprisingly, when we performed saturation-binding EMSA experiments using increasing amounts of a DNA probe containing only one CArG-box, the mutant protein SEP3-E161L-N168L exhibited no binding of individual dimers. Instead, a signal of low electrophoretic mobility occasionally occurred for high amounts of applied DNA probe, which might constitute a protein–DNA complex consisting of more than two proteins (Supplementary Fig. S4).

### Mutations in the most-distantly related ortholog of SEP3 have very similar effects on co-operative DNA-binding as in SEP3

Next, we aimed to assess whether the importance of the identified leucine residues is evolutionarily conserved within the SEP3 subfamily. The MADS-domain TF AMtrAGL9 from *Amborella trichopoda* constitutes the most-distantly related ortholog of SEP3 (Zahn *et al.*, 2005). In EMSA experiments, AMtrAGL9 forms homotetrameric protein–DNA complexes with a co-operative binding affinity comparable to SEP3 (Fig. 2E, Supplementary Fig. S5). AMtrAGL9 amino acid position I141 is homologous to SEP3 L145 and is thus located in the K2-subdomain heptad-repeat of AMtrAGL9 (Fig. 2A). Substitution to alanine at that position interfered to some extent with co-operative binding capabilities, whereas substitution to proline at position I141 resulted in an almost complete loss of co-operative binding (Fig. 2E, Supplementary Table S1). If the amino acid position L160 of AMtrAGL9, which is homologous to position L164 in the center of the K3-subdomain of SEP3, was exchanged by proline or alanine, the ability of AMtrAGL9 to co-operatively bind to DNA was almost completely lost in either case, a behavior that was similar to that observed for SEP3 (compare Fig. 2D and E).

### Interacting sites are more often occupied by leucine in SEP3-subfamily proteins than in proteins of other MIKC-type subfamilies

The importance of leucine residues for the tetramerization ability of SEP3 and AMtrAGL9 raised the question as to what extent these positions are conserved within the SEP3 subfamily, and which amino acid preferences members of other MIKC-type protein subfamilies show at homologous sites. We therefore created a multiple sequence alignment based on 1325 sequences of MIKC-type MADS-domain proteins belonging to 14 subfamilies and comprising sequences from a diverse array of seed plants. Despite the high evolutionary distance of the sampled taxa, the sequences aligned almost without gaps throughout the complete K-domain (i.e. without potential insertions or deletions). After exclusion of a few sequences that produced gaps and that appeared not to be representative for the respective subfamily, the only gap was produced by PI-subfamily protein sequences, among which a deletion of four amino acids within the C-terminal half of the K-domain was very common. This deletion within the PI-linage most likely occurred after early-diverging angiosperms branched off, as most of the sampled PI-subfamily sequences from early-diverging angiosperms still possessed those four amino acids.

**Fig. 3.**
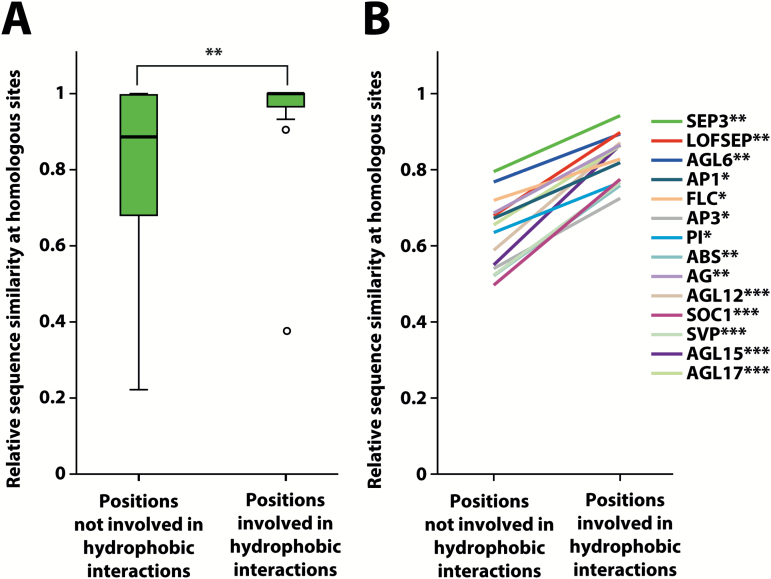
Sequence similarity analysis of SEP3-subfamily proteins and members of other MIKC-type MADS-domain protein subfamilies. (A) Box-plot showing relative sequence similarity at homologous sites of SEP3-subfamily proteins for positions that are involved in hydrophobic interactions within the SEP3 homotetramer and positions that are not involved in hydrophobic interactions. (B) Graph showing the same analysis as in (A) but for all MIKC-type protein subfamilies. For all subfamilies, amino acid positions that are homologous to sites involved in hydrophobic interactions are significantly less variable than positions that are homologous to non-interacting sites (Mann–Whitney *U*-test; *P<0.05; **P<0.01; ***P<0.001).

We first compared the conservation of sites that are homologous to the 15 residues that (based on the crystal structure of SEP3) mediate the hydrophobic intra- and intermolecular interactions in the SEP3 homotetramer (Puranik *et al.*, 2014) to the overall conservation of the K-domain. We found that within the SEP3 subfamily, sites that were homologous to interacting sites in the SEP3 homotetramer were significantly less variable than the remaining residues of the K-domain (Fig. 3A). This conservation pattern also held true for sequences of all the other 13 subfamilies of MIKC-type MADS-domain proteins that we examined (Fig. 3B, Supplementary Fig. S6A), as well as for sequences from gymnosperms to core eudicots (see Supplementary Fig. S6B). Beyond this similar pattern of conserved positions, the amino acid properties in terms of hydrophobicity at homologous sites also appeared highly similar among all the examined subfamilies (Supplementary Fig. S7), suggesting that the overall structure of the K-domain as determined for SEP3 is conserved among MIKC-type proteins of most, if not, all subfamilies and throughout seed plants.

**Fig. 4.**
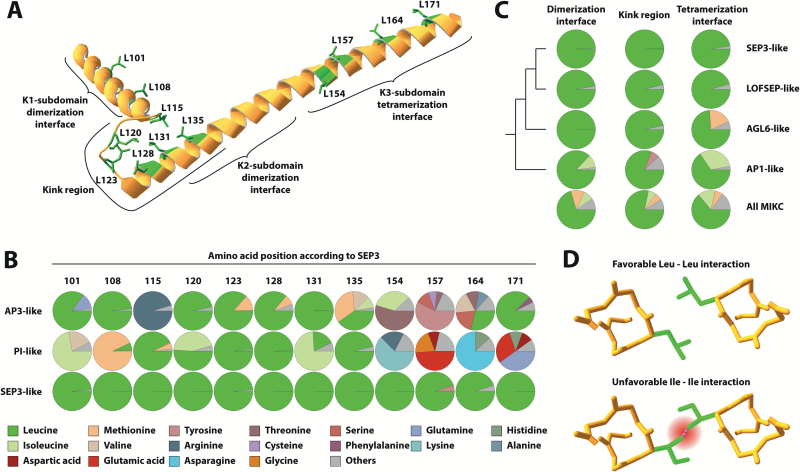
Amino acid preferences of SEP3-subfamily proteins and members of other MIKC-type MADS-domain protein subfamilies. (A) Picture of the crystal structure of a single K-domain of SEP3. Leucine side chains that are involved in inter- and intramolecular interactions are shown in green. (B) Amino acid frequencies at sites homologous to leucine residues that are involved in inter- and intramolecular interactions in the SEP3 homotetramer, shown for SEP3-, AP3-, and PI-subfamily proteins. Amino acids that occurred in less than 5% of the examined subset of sequences are condensed as ‘others’. The vast majority of the positions shown vertically are homologous to each other. The only exceptions are positions 154, 157, 164, and 171 of PI-like proteins. In this case, a gap was detected in the alignment but amino acids directly following the gap are included here. (C) Amino acid preferences at sites homologous to leucine residues that contribute to the dimerization interface (L101, L108), kink region (L115, L120, L123, L128, L131, L135), and tetramerization interface (L154, L157, L164, L171) in the SEP3 homotetramer, shown for SEP3-, LOFSEP-, AGL6-, and AP1-subfamily proteins and all MIKC-type proteins, following the same color-coding in (B). (D) Part of the crystal structure of two interacting tetramerization interfaces within a SEP3 homotetramer. The picture illustrates the favorable Leu–Leu interaction at heptad-repeat ‘d’ positions that occurs several times within a SEP3 homotetramer (upper part). In contrast to the γ-branched leucine, a β-branched amino acid such as isoleucine would potentially lead to steric hindrance at heptad-repeat ‘d’ positions (lower part).

Next, we analysed the amino acid distribution at sites homologous to the 12 leucine residues (L101, L108, L115, L120, L123, L128, L131, L135, L154, L157, L164, and L171) that contribute to inter- and intramolecular interactions in a SEP3 homotetramer (Fig. 4A) (Puranik *et al.*, 2014). Despite the high evolutionary distance of the examined SEP3-subfamily proteins (the alignment included sequences from *Amborella*, Nymphaeales, monocots, and eudicots), all these leucine residues were found to be highly conserved within the 78 examined sequences; 8 out of 12 positions were completely invariable (Fig. 4B). In contrast to this, members of other subfamilies (e.g. AP3- and PI-subfamily proteins) often showed preferences for other amino acids on equivalent sites (Fig. 4B, Supplementary Fig. S8). In particular, positions equivalent to L154, L157, and L164 of SEP3 that are located within the center of the tetramerization interface were often not occupied by leucines in AP3- and PI-subfamily proteins. The conservation of leucines was also very high within LOFSEP-subfamily proteins (comprising SEP1, SEP2, and SEP4 from *A. thaliana*), although not as high as in the SEP3 subfamily. LOFSEP proteins form the sister group of SEP3-subfamily proteins and are assumed to function in a mostly redundant manner with SEP3 during flower development (Fig. 4C) (Pelaz *et al.*, 2000; Ditta *et al.*, 2004; Zahn *et al.*, 2005). The closest relatives of SEP3- and LOFSEP-subfamily proteins are AGL6-subfamily proteins, followed by AP1-subfamily proteins (Kim *et al.*, 2013). However, despite this close relationship, AGL6- as well as AP1-subfamily proteins displayed a considerably lower leucine frequency, especially on sites within the tetramerization interface (Fig. 4C, Supplementary Fig. S8). Instead, these positions were more frequently occupied by other hydrophobic amino acids, such as isoleucine and methionine. It has previously been shown that within a coiled-coil, leucine packs very well at heptad-repeat ‘d’ positions and enables the formation of a tight dimeric coiled-coil as it becomes apparent in a leucine-zipper (Zhu *et al.*, 1993; Betz *et al.*, 1995). In contrast, other hydrophobic amino acids such as isoleucine or valine lead to steric hindrance at heptad-repeat ‘d’ positions (Betz *et al.*, 1995) (Fig. 4D).

### Insertion of leucine residues into the K3-subdomain of AP3 facilitates homotetramerization of the chimeric protein SEP3_AP3chim_

**Fig. 5.**
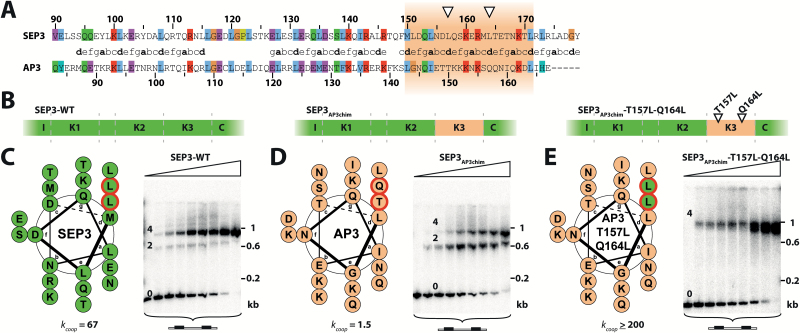
Design and co-operative DNA-binding capabilities of the chimeric protein SEP3_AP3chim_. (A) Pairwise sequence alignment of the K-domains of SEP3 and AP3; the heptad-repeat pattern is depicted between the two. The orange background marks the region that was substituted to create the chimeric protein SEP3_AP3chim_. The triangles mark the positions of the subsequently introduced amino acid substitutions. (B) Experimental set-up to test for the ability of leucines to restore tetramerization ability of SEP3_AP3chim_. First, the complete tetramerization interface (i.e. the K3-subdomain) of SEP3 was substituted by the equivalent positions of AP3. Subsequently, the two residues T157 and Q164 were substituted back to leucine to re-establish the hydrophobic stripe. (C–E, left) Helical wheel diagrams of the tetramerization interface of SEP3 wild-type (C), SEP3_AP3chim_ (D), and SEP3_AP3chim_-T157L-Q164L (E), to illustrate the presumed positions of amino acids 157 and 164 (circled in red) within the hydrophobic stripe of the K3-subdomain coiled-coil. (C–E, right) Binding of SEP3 wild-type (C), SEP3_AP3chim_ (D), and SEP3_AP3chim_-T157L-Q164L (E) to a DNA probe containing two CArG-boxes. Increasing amounts of *in vitro* translated protein were incubated together with constant amounts of DNA probe. *k*_coop_ values inferred from this particular measurement are given below the helical wheel diagrams. The low *k*_coop_ value of SEP3_AP3chim_ indicates that co-operative binding was almost abolished. The bands of low electrophoretic mobility (labelled with ‘4’) most likely represent two dimers bound independently of each other to the two CArG-boxes on the DNA probe.

Based on our data, we hypothesized that the overall structure of the K-domain is conserved throughout most, if not all, subfamilies of MIKC-type MADS-domain proteins and that evolutionarily conserved preferences for different amino acids on interacting sites account for subfamily-specific interaction capabilities. We aimed to test our hypothesis with help of the chimeric protein SEP3_AP3chim_, in which we substituted the K3-subdomain (i.e. tetramerization interface) of SEP3 (residues 150–181) by the homologous sites of AP3 (Fig. 5A, B). AP3 is known to form obligate heterodimers with PI and is thus not able to form DNA-binding homodimers or homotetramers (Riechmann *et al.*, 1996; de Folter *et al.*, 2005; Melzer and Theissen, 2009). As expected, the chimeric protein SEP3_AP3chim_ showed a complete loss of homotetramerization capabilities in EMSA experiments compared to the SEP3 wild-type protein (Fig. 5C, D, Supplementary Table S1), but it retained the ability to bind as a dimer to DNA. Although the K3-subdomains of SEP3 and AP3 share only four identical residues at homologous sites, the sequence similarity in terms of hydrophobicity on most heptad-repeat ‘a’ and ‘d’ positions was comparatively high (Fig. 5A). However, two heptad-repeat ‘d’ positions occupied by leucine in SEP3 (L157 and L164) were occupied by threonine and glutamine in AP3, respectively (Fig. 5A). Both leucines were highly conserved throughout SEP3-subfamily proteins, whereas homologous sites in AP3-subfamily proteins were almost exclusively occupied by residues other than leucine (Fig. 4B). We thus substituted positions T157 and Q164 of the chimeric protein by leucine and tested the ability of the resulting mutants to form homotetramers. Both single-amino acid substitutions could not improve the tetramerization ability of the chimeric protein (see Supplementary Table S1). However, the insertion of both leucine residues into the K3-subdomain of SEP3_AP3chim_ sufficed to fully restore the ability to form DNA-binding homotetramers (Fig. 5E, right panel). Visualizing the amino acid sequence of the tetramerization interface of SEP3 and AP3 in a helical wheel diagram illustrates how the residues M150, L157, L164, and L171 form a strong hydrophobic stripe within the tetramerization interface of SEP3, whereas the hydrophobic stripe is interrupted by threonine and glutamine in AP3 (Fig. 5C, D left panels). Substituting both residues by leucine closes the gap within the hydrophobic stripe and thereby probably facilitates homotetramerization (Fig. 5E, left panel).

## Discussion

### Conserved leucine residues in the K-domain of SEP3 are required for tetramer formation

Tetramer formation among MIKC-type MADS-domain transcription factors is of central importance for flower development (Theissen and Saedler, 2001; Immink *et al.*, 2009; Melzer and Theissen, 2009; Smaczniak *et al.*, 2012; Theißen *et al.*, 2016). However, knowledge about the molecular determinants facilitating tetramer formation remained scarce. Our data indicated that substitution of leucines in the K-domain of SEP3 almost invariably led to a strong reduction in tetramer formation abilities (Fig. 2). This was expected for leucine-to-proline substitutions within the helical regions of the K-domain as proline has helix-breaking properties. However, the rather conservative substitution from leucine to alanine in the tetramerization interface (L164A) also strongly affected co-operative binding and tetramerization. Similar results have been obtained for substituting other leucine residues in the tetramerization interface by alanine (Puranik *et al.*, 2014).

The question arises as to why specifically leucine residues are favoured over other hydrophobic amino acid residues. The tetramerization interface forms coiled-coils and it is well established that complex ‘knobs-into-holes’ side-chain interactions within the hydrophobic core determine the strength of the interaction between coiled-coils (Parry *et al.*, 2008). Numerous studies on energetic contributions of different hydrophobic amino acids inside the hydrophobic core have revealed that β-branched amino acids (e.g. isoleucine or valine) as well as amino acids with small side-chains (e.g. alanine) in heptad-repeat ‘d’ positions have a strong destabilizing effect on the formation of parallel dimeric coiled-coils (Zhu *et al.*, 1993; Takei *et al.*, 2006). The local stereochemical environment at heptad-repeat ‘d’ positions instead strongly favours γ-branched amino acids for intermolecular interactions, making leucines uniquely suited at these sites (Zhu *et al.*, 1993; Betz *et al.*, 1995; Moitra *et al.*, 1997; Takei *et al.*, 2006). This is in line with the observation that L145, which is located at a heptad-repeat ‘d’ position but according to structural data is not involved in intermolecular interactions (Puranik *et al.*, 2014), can be mutated to alanine without a decrease in tetramer formation capabilities. In contrast, mutation of L164 (also at a heptad-repeat ‘d’ position but involved in intermolecular interactions) to alanine leads to a strong decrease in tetramerization. In addition, L145 is not nearly as conserved as leucines involved in interactions (see Supplementary Fig. S8).

A decrease in tetramer formation was also observed for substitution of leucines in the kink region between the two helices, where an effect on helix formation was not predicted (see Supplementary Fig. S1). However, although the leucine residues in the kink are not directly involved in tetramer formation, they interact intramolecularly with each other to stabilize the kink and thus bring the tetramer interface into a favourable position for protein–protein interactions (Puranik *et al.*, 2014). It is likely that substitutions to proline or alanine in the kink region altered or destabilized the orientation of the tetramerization interface and thus impeded tetramer formation indirectly. Similar to the leucines at interacting sites within the helical regions of the K-domain, stereochemical restrictions may also in this case favour leucines over other hydrophobic amino acids. This may explain why the L115A mutation in the kink region, which presumably only affects intramolecular interactions, caused a decrease in tetramer formation capabilities.

Taken together, these findings indicate that inter- and intramolecular hydrophobic interactions specifically among leucines are of critical importance for SEP3 homotetramerization. This principle very likely applies to the entire subfamily of SEP3-like proteins, as leucines at interaction positions are evolutionarily highly conserved throughout this subfamily (Fig. 4). The evolutionarily conserved and important role of leucines is further highlighted by the observation that in the SEP3 ortholog AMtrAGL9 from *A. trichopoda*, leucines at positions homologous to those in SEP3 were also of critical importance for tetramer formation (Fig. 2).

In a recent study, Ruelens *et al.* (2017) reconstructed and synthesized the putative ancestral SEP3 sequence present at the base of angiosperm evolution. Interestingly, the reconstructed sequence carries all the leucine residues that constitute interaction sites within the K-domain of SEP3. Given this presence of leucine residues, the ancestral SEP3 sequence seems to be capable of forming floral quartet-like complexes and mediating the interaction of other ancestral floral homeotic proteins (Ruelens *et al.*, 2017).

### Structural similarity and interaction specificity among the K-domains of MIKC-type proteins

The K-domain is the second highest-conserved domain of MIKC-type proteins (the most-highly conserved being the MADS-domain) (Kaufmann *et al.*, 2005). Previous structural predictions indicated that the K-domain forms coiled-coils in most, if not all, MIKC-type proteins (Ma *et al.*, 1991; Riechmann and Meyerowitz, 1997; Puranik *et al.*, 2014). Indeed, our analyses strongly supported this view. The chemical properties of amino acids that are of importance for intra- and intermolecular interactions in SEP3 were conserved in MIKC-type proteins from all of the 14 subfamilies analysed here. This indicates that most K-domains fold into a structure similar to that determined for SEP3, and that residues that are homologous to interacting sites in the SEP3 homotetramer may also constitute intra- and intermolecular contact points in most other protein family members.

However, although the chemical properties of amino acids important for interactions were conserved in subfamilies other than SEP3, this was not always the case for the amino acid identities. Whereas the vast majority of leucine residues important for intra- and intermolecular interactions were highly conserved within the SEP3 subfamily, they were observed at a noticeably lower frequency in other subfamilies (Fig. 4, Supplementary Fig. S4). This indicates that, although the overall structure of the K-domain is conserved in all MIKC-type proteins and probably throughout angiosperm evolution, the tetramerization capabilities of MIKC-type proteins may vary depending on the presence of leucines on critical interaction sites. For example, AP3 and PI, which do not possess leucines on all inter- and intramolecular contact points, are unable to form tetramers not involving SEP3 (Melzer and Theissen, 2009; Smaczniak *et al.*, 2012). Indeed, the K3-subdomain of AP3, which is not capable of mediating homotetramer formation, gained this ability when placed in the SEP3 protein context and two leucines were introduced (Fig. 5). Thus, we hypothesize that leucines at intra- and intermolecular contact points may not only be necessary but also sufficient for tetramer formation of MIKC-type proteins.

### The ability of SEP3-subfamily proteins to act as hub proteins may depend on highly conserved leucines

Whether homotetramers of SEP3 or MIKC-type proteins in general have a biological function has not been demonstrated yet, but neither can the possibility be excluded (for a more detailed discussion, see Melzer *et al.*, 2009). Intriguingly, the high conservation of leucines in the K-domain of SEP3-subfamily proteins and their importance for homotetramer formation correlates very well with the crucial function of these proteins as hubs within the protein–protein interaction network that controls flower development. In addition, proteins such as AP3 and PI that have less central roles within the interaction network also lack leucines at several positions critical for tetramerization. It thus appears plausible that leucines in SEP3-subfamily proteins are not only important for homotetramer formation but also play a pivotal role in the formation of heterotetrameric complexes. For example, although a lack of leucines in the kink region of many MIKC-type proteins may destabilize the orientation of the tetramerization interface and prevent homotetramer formation, the high structural stability of the K-domain of SEP3-subfamily proteins that is brought about by intramolecular leucine interactions may serve as a scaffold that helps to align the interaction interface of partner proteins and hence facilitates heterotetramer formation.

The pattern of leucines at the tetramerization interface may be explained in a similar manner. Although data on the interaction of leucines at heptad-repeat ‘d’ positions with other amino acids at ‘d’ positions in a heteromeric coiled-coil are scarce, data from leucine-zippers indicate that beyond leucine–leucine interactions, interactions of leucines with a number of other amino acids are more favourable than most other interactions that do not involve any leucine (Fong *et al.*, 2004).

Taken together, we propose that the leucine residues in SEP3-subfamily proteins serve to facilitate heterotetrameric interactions, while at the same time the absence of leucines in the interaction partners prevents homotetramer formation or the formation of heterotetramers not involving SEP3-subfamily proteins. This way, tetramerization of many MADS-domain transcription factors depends on the presence of SEP3-subfamily proteins and close relatives (e.g. LOFSEP proteins) and probably cannot occur without them.

## Conclusions and outlook

We have previously proposed that the dependence of other MIKC-type proteins on SEP3- and LOFSEP-subfamily proteins for tetramer formation facilitated the concerted development of the different floral organs and the evolution of the flower as a single reproductive entity (Melzer *et al.*, 2014). The evolutionary conservation of leucines in the SEP3 subfamily as opposed to most other subfamilies may thus be one of the important molecular mechanisms that fostered the evolution of the flower.

It is important to note, however, that coiled-coil interactions are very complex, with the amino acids that occupy the heptad-repeat ‘a’, ‘d’, ‘e’, and ‘g’ positions playing key roles in determining the affinity and specificity of an interaction (Mason and Arndt, 2004; Mason *et al.*, 2009; Potapov *et al.*, 2015), and we are far from completely understanding the implications for MIKC-type protein interactions of sequence variations on the different positions. For example, polar and charged residues are observed at heptad-repeat ‘d’ positions in a number of MIKC-type protein subfamilies and they would be expected to not only hinder homotetramerization but also heterotetramerization with SEP3-subfamily proteins. Similarly, our observation that the introduction of additional leucines can increase co-operativity (e. g. in the E161L-N168L mutant) as compared to the wild-type protein may be taken as indication that *in vivo* co-operative DNA-binding is a finely tuned system that is balanced between stable DNA-binding whilst still maintaining flexibility for regulatory input and interaction with different partner proteins. Furthermore, subfamily-specific patterns of charged residues at heptad-repeat ‘e’ and ‘g’ positions can be observed that would be expected to contribute to interaction specificity. These charge distribution patterns could probably explain why heterotetramers are usually formed in favour of homotetramers. Although our findings bring us a step closer towards solving the code for floral quartet-like complex formation, additional structural, biophysical and, importantly, *in vivo* analyses are required to more completely understand the molecular mechanisms and evolutionary patterns of MIKC-type protein interactions. This will eventually also lead to a better understanding as to why this transcription factor family expanded in seed plants and why it plays a role in virtually every reproductive developmental process.

## Supplementary data

Supplementary data are available at *JXB* online.

Table S1. Summary of all the examined SEP3 and AMtrAGL9 constructs.

Fig. S1. Coiled-coil predictions for SEP3 wild-type protein, all single and double proline-substitution mutants, and SEP3-E161L-N168L.

Fig. S2. Dimer binding affinity of SEP3 and AMtrAGL9 wild-type and mutant proteins.

Fig. S3. Comparison of the ability of SEP3 wild-type, SEP3-L145A, and SEP3-E161L-N168L to co-operatively bind to DNA.

Fig. S4. Comparison of the binding behaviour of SEP3-E161L-N168L and SEP3-G178P to a DNA probe containing a single CArG-box.

Fig. S5. Ability of AMtrAGL9 wild-type protein to co-operatively bind to DNA.

Fig. S6. Sequence similarity analysis for all subfamilies of MIKC-type MADS-domain proteins and different organism groups.

Fig. S7. Comparison of hydrophobicity patterns within the K-domain for all subfamilies of MIKC-type MADS-domain proteins.

Fig. S8. Analysis of amino acid composition of the K-domain for all MIKC-type protein subfamilies.

Supplementary Data
