# Supplementary material for: A conserved leucine zipper-like motif accounts for strong tetramerization capabilities of SEPALLATA-like MADS-domain transcription factors
Source: J Exp Bot. 2018 Feb 21;69(8):1943–54. doi: 10.1093/jxb/ery063 (PMC6018978; doi:10.1093/jxb/ery063)
Supplement: Supplementary Data [file ery063_suppl_supplementary_data.pdf]

Supplementary Table S1. Summary of all examined SEP3 and AMtrAGL9 constructs. For each of the examined constructs the location of the substitution site, the frequency of the initial amino acid within the SEP3 subfamily as well as among all MIKC-type proteins, the measured dimerization ability and the inferred ability to form tetrameric complexes is shown (each number constitutes an individual replicate). Amino acid frequencies were calculated based on the multiple sequence alignment of 1325 MIKC-type proteins described in Methods. The frequency of glycine at position 178 among all examined MIKC-type proteins could not be determined as this part of the sequences did not align properly. For the mutant protein SEP3-E161L-N168L no dissociation constant for binding of a protein dimer to a single CArG-box (k_d) could be determined because no binding of individual dimers occurred. k_{coop} values above 200 could not be determined reliably (see Methods).

	Construct	Location of substitution site	Frequency of initial amino acid		Dimerization ability (k_d in nM)				Tetramerization ability (k_{coop})						
			Within SEP3 subfamily	Among all MIKC-type proteins	1	2	3	4	1	2	3	4	5	6	7
SEP3 constructs	WT	-	-	-	4.4 ± 0.5	6.2 ± 0.8			54.0	≥200	≥200	67.3	119.4	≥200	150.3
	S94P	K1	96.2 %	10.2 %	5.1 ± 0.8	6.3 ± 0.9	5.7 ± 0.5		≥200	133.4	≥200				
	L115P	Kink	100 %	57.4 %	6.1 ± 1.2	2.4 ± 0.4			17.3	40.2	22.4				
	L115A				9.8 ± 0.8	1.7 ± 0.6	3.2 ± 0.7		23.9	3.9	1.8				
	L120P-L123P	Kink	100 % / 98.7 %	83.4 % / 71.7 %	6.2 ± 1.9	5.7 ± 0.7	6.6 ± 1.3		5.4	5.8	7.4				
	L131P-L135P	Kink	100 % / 100 %	78.1 % / 84.7 %	3.3 ± 1.2	1.8 ± 0.4	5.5 ± 1.6		3.7	18.9					
	L145P	K2	5.1 %	0.3 %	8.0 ± 2.2	1.8 ± 0.3	4.9 ± 1.5		3.6	3.8	17.1				
	L145A				3.1 ± 0.4	0.9 ± 0.2	3.5 ± 1.0		≥200	≥200	≥200				
	L164P	K3	94.9 %	62 %	4.4 ± 1.0	4.4 ± 1.5	1.5 ± 0.2		1.8	4.3	2.8	15.4	2.4		
	L164A				5.2 ± 1.3	1.6 ± 0.2			9.2	2.9	3.7	3.3			
	L164E				4.2 ± 0.9	2.0 ± 0.5			1.6	2.5	2.3	1.3			
	L164W				8.6 ± 1.7	4.2 ± 0.5	6.8 ± 1.6	5.8 ± 1.2	17.7	72.8	4.2	7.2			
	L164I				7.6 ± 2.2	5.1 ± 0.8			18.2	4.4	3.2				
	E161L-N168L	K3	100 % / 100 %	68.3 % / 71.2 %	no dimer binding				≥200	≥200	≥200	≥200			
	G178P	K3	64.1 %	Uncertain homology assignment	5.3 ± 1.2	1.6 ± 0.3			47.8	124.7	≥200				
	$SEP3_{AP3chim}$	K3	-	-	5.5 ± 1.8				2.6	11.5	1.5				
	$SEP3_{AP3chim}$-T157L	K3	0 %	1.7 %	7.4 ± 2.3				3.2	1.8	16.3				
	$SEP3_{AP3chim}$-Q164L	K3	0 %	1 %	6.2 ± 0.7				1.4	1.7	3.6				
	$SEP3_{AP3chim}$-T157L-Q164L	K3	0 % / 0 %	1.7 % / 1 %	2.9 ± 1.2				≥200	136.1					
AMtrAGL9 constructs	WT	-	-	-	3.3 ± 0.7				≥200	123.3	121.0				
	I141P	K2	6.4 %	2.1 %	1.2 ± 0.3				3.8	5.4					
	I141A				4.1 ± 1.0				39.1	28.8					
	L160P	K3	94.9 %	62 %	4.1 ± 0.7				2.1	1.3	1.8	1.6	2.5		
	L160A				3.4 ± 0.9				1.9	2.4	2.5	1.4			

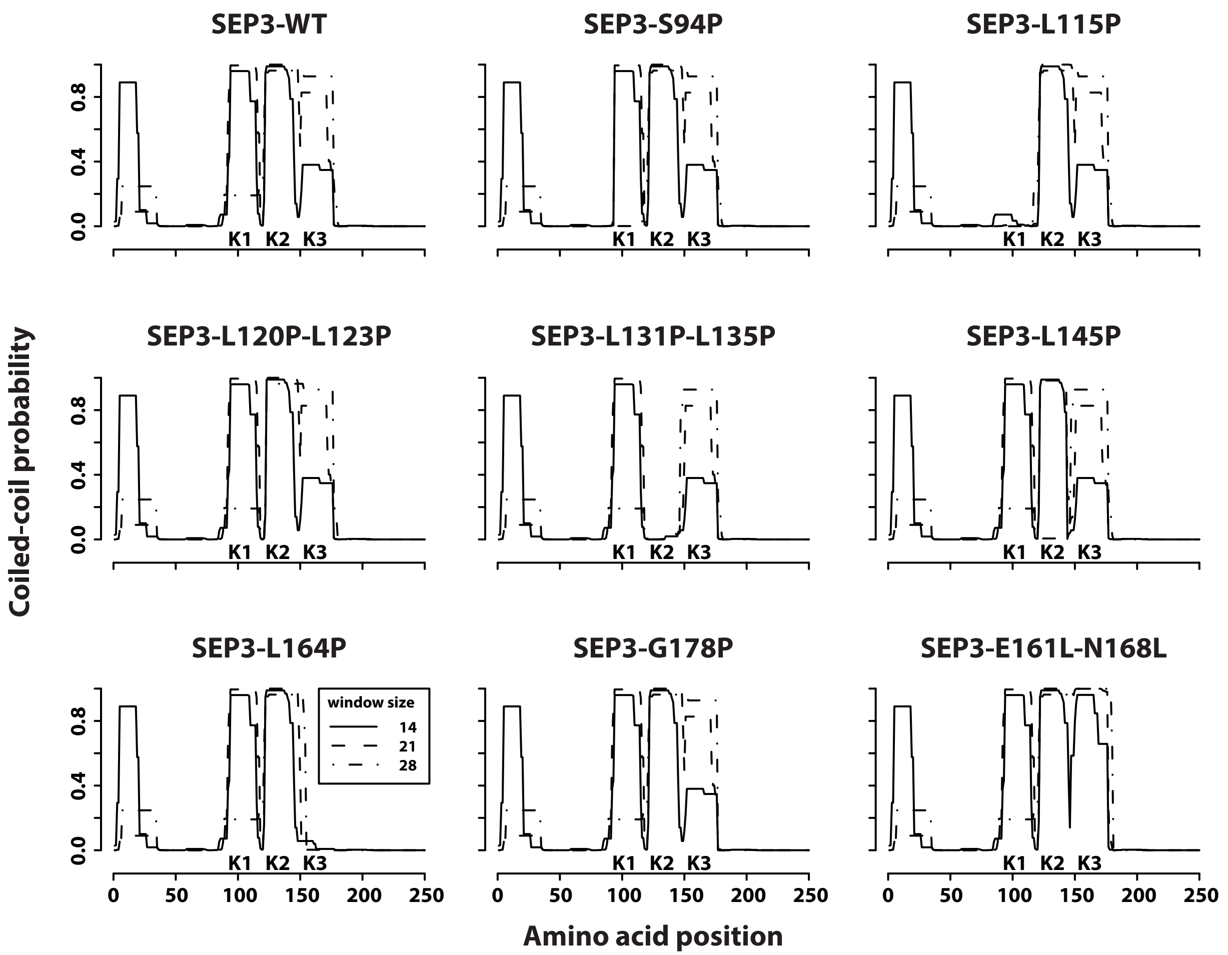

Supplementary Figure S1. Coiled-coil predictions for SEP3 wild type protein, all single and double proline substitution mutants and SEP3-E161L-N168L. Coiled-coil probability values were calculated with COILS (Lupas et al., 1991) and visualized using R. The solid, dashed and dotted lines correspond to a sliding window size of 14, 21 and 28 amino acids used for the prediction, respectively.

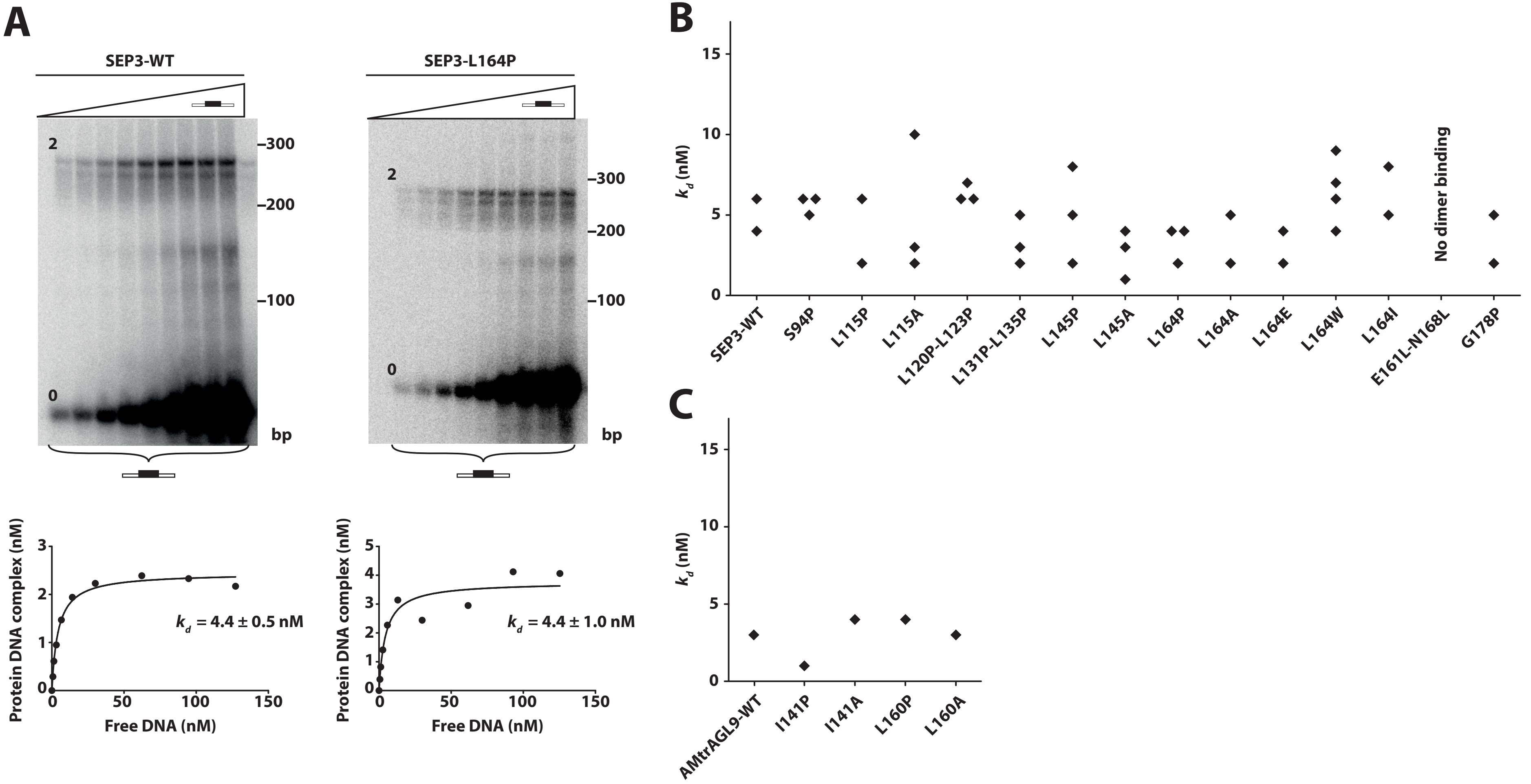

Supplementary Figure S2. Dimer binding affinity of SEP3 and AMtrAGL9 wild type and mutant proteins. (A) To estimate absolute values for the dissociation constant for binding of a protein dimer to a single DNA-binding site k_d, saturation binding assays were performed, using a constant amount of protein (2-5 µl, depending on the estimated *in vitro* translation efficiency of the respective construct) together with increasing amounts of a DNA probe carrying a single CArG-box. The amounts of DNA used for the binding reactions were 0.25, 0.5, 1, 2, 4, 8, 16, 24, and 32 ng. The labeling of the two different fractions '0' and '2' corresponds to the number of proteins bound to one DNA molecule. It is not entirely clear why two protein-DNA-complexes were observed here, but similar observations were made previously (Melzer et al., 2014). Signal intensities of the different fractions were quantified and the amount of protein-DNA complex was plotted as a function of the amount of free DNA as shown below the gel pictures. The saturation curve was fitted according to equation (5) described in Methods and as previously described (Jetha et al., 2014). The k_d value inferred from this particular measurement is depicted in the diagram. (B and C) kd values for SEP3 (B) and AMtrAGL9 (C) wild type and mutant proteins, respectively. For the mutant protein SEP3-E161L-N168L no binding of individual dimers was observed (see main text and Supplementary Figure 4).

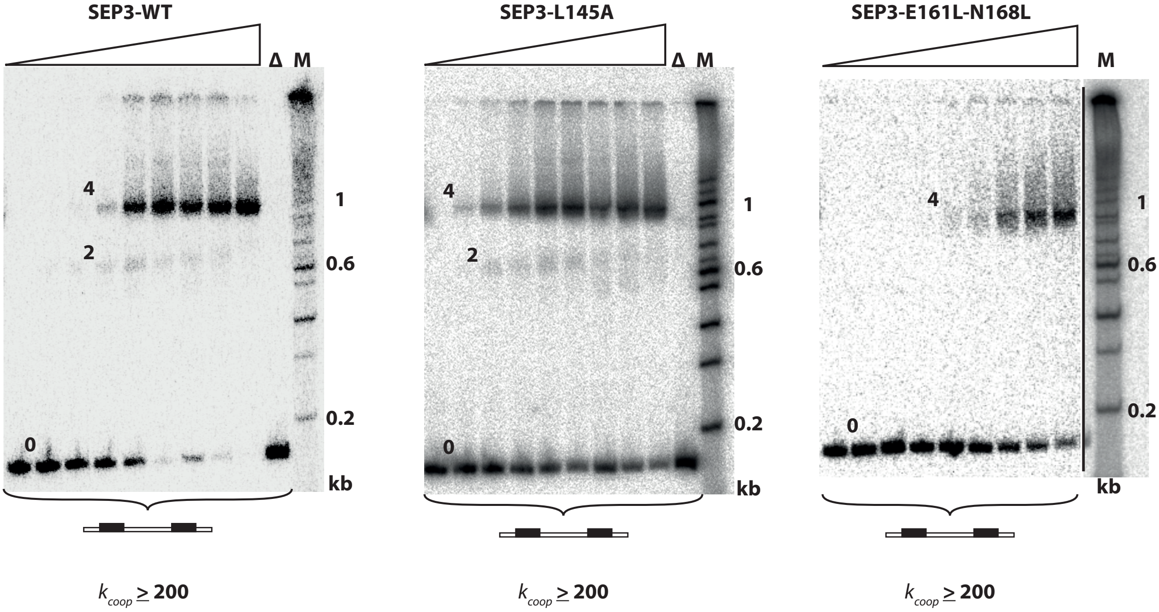

Supplementary Figure S3. Comparison of the ability of SEP3 wild type (left), SEP3-L145A (middle) and SEP3-E161L-N168L (right) to cooperatively bind to DNA. Increasing amounts of *in vitro* translated protein were incubated with constant amounts of DNA probe. The volumes of *in vitro* translated protein used for the binding reactions were 0, 0.05, 0.1, 0.2, 0.4, 0.6, 1, 1.5 and 3 µl. As negative control the DNA probe was incubated together with 3 µl of *in vitro* translation solution for which the empty pTNT vector without any cDNA insert was used as template DNA (lane Δ). For size comparison a radioactively labeled DNA ladder (100 bp Ladder, NEB) was applied (lane M). The labeling of the three different fractions '0', '2' and '4' corresponds to the number of proteins bound to one DNA molecule. The k_{coop} value inferred from this particular measurement is depicted below the gel picture.

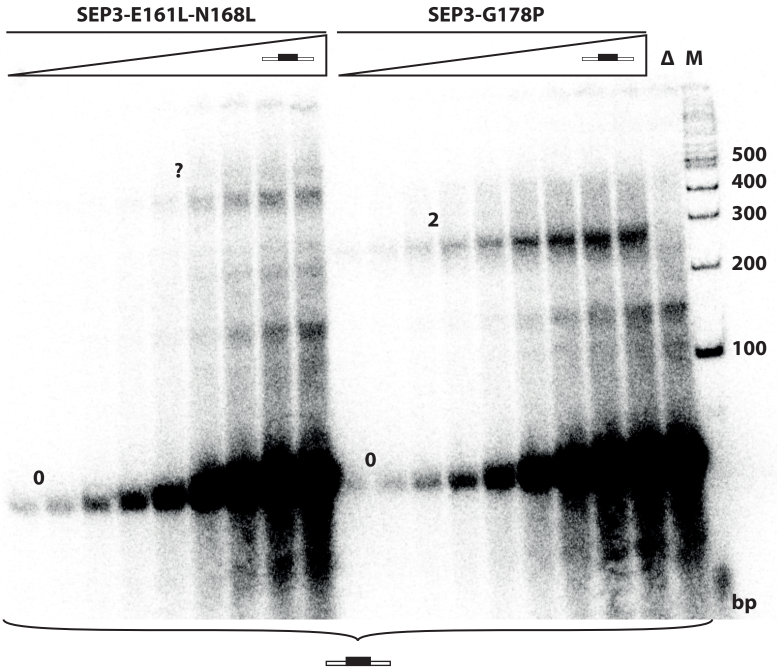

Supplementary Figure S4. Comparison of the binding behavior of SEP3-E161L-N168L (left) and SEP3-G178P (right) to a DNA probe containing a single CArG-box. Experimental details are described in the legend to Supplementary Figure 2. As negative control 32 ng DNA probe were incubated together with 2 µl of *in vitro* translation solution for which the empty pTNT vector without any cDNA insert was used as template DNA (lane Δ). For size comparison a radioactively labeled DNA ladder (100 bp Ladder, NEB) was applied (lane M). The labeling of the different fractions '0' and '2' corresponds to the number of proteins bound to one DNA molecule. The question mark '?' denotes a protein-DNA complex of unknown stoichiometry. In contrast to SEP3 wild type protein and all other SEP3 mutants, SEP3-E161L-N168L produced no band that migrates with the velocity of a DNA probe bound by two proteins, as it is exemplarily shown for SEP3-G178P. Instead a band of low electrophoretic mobility occurs for high amounts of applied DNA probe.

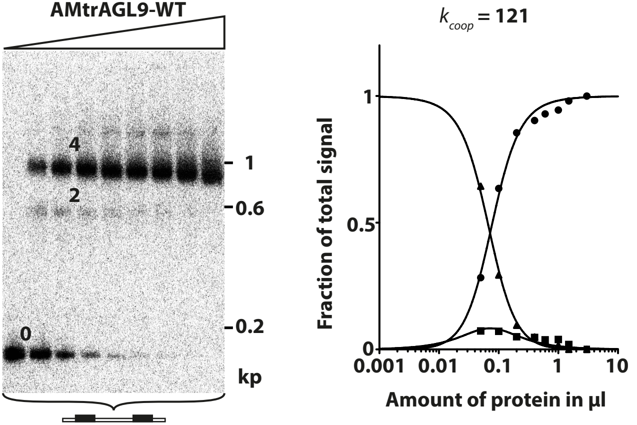

Supplementary Figure S5. Ability of AMtrAGL9 wild type protein to cooperatively bind to DNA. Binding of AMtrAGL9 to a DNA probe containing two CArG-boxes in a distance of six helical turns. Increasing amounts of *in vitro* translated protein were incubated with constant amounts of DNA probe. The volumes of *in vitro* translated protein used for the binding reactions were 0, 0.05, 0.1, 0.2, 0.4, 0.6, 1, 1.5 and 3 µl. The labeling of the three different fractions '0', '2' and '4' corresponds to the number of proteins bound to one DNA molecule. Quantified signal intensities of the different fractions are shown next to the gel picture (triangle: free DNA; square: DNA probe bound by two proteins; circle: DNA probe bound by four proteins). Graphs were fitted according to equation (1) to (3) described in Methods and as previously described (Senear and Brenowitz, 1991; Melzer et al., 2009). The k_{coop} value inferred from this particular measurement is depicted above the diagram.

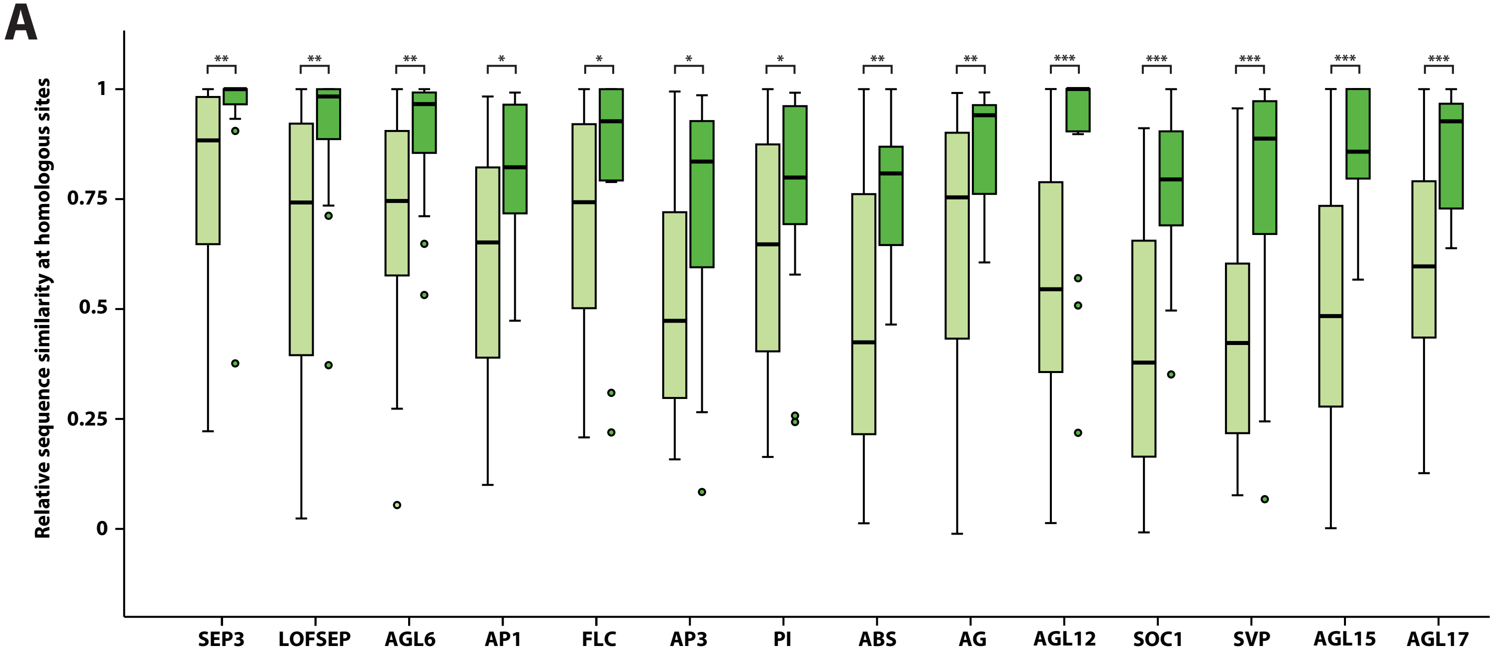

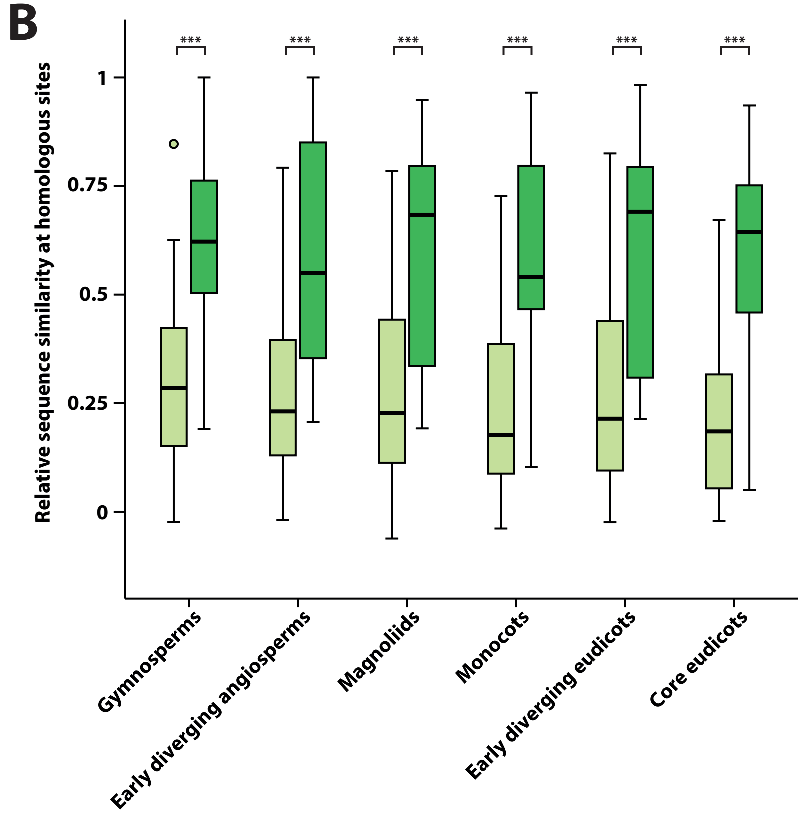

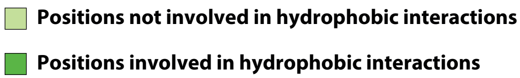

Supplementary Figure S6. Sequence similarity analysis for all subfamilies of MIKC-type MADS-domain proteins and different organism groups. Box-plots depicting relative sequence similarities at homologous sites for positions that are involved in hydrophobic interactions within the SEP3 homotetramer and positions that are not involved in hydrophobic interactions shown for all 14 subfamilies of MIKC-type MADS-domain proteins (A) and for sequences from gymnosperms, early diverging angiosperms, magnoliids, monocots, early diverging eudicots and core eudicots (B). For all MIKC-type protein subfamilies as well as for the 6 examined organism groups amino acid positions that are homologous to sites involved in hydrophobic interactions in the SEP3 homotetramer are significantly less variable than positions that are homologous to non-interacting sites (Mann-Whitney-U-test; * $p = 0.01$-0.05; ** $p = 0.001$-0.01; *** $p < 0.001$).

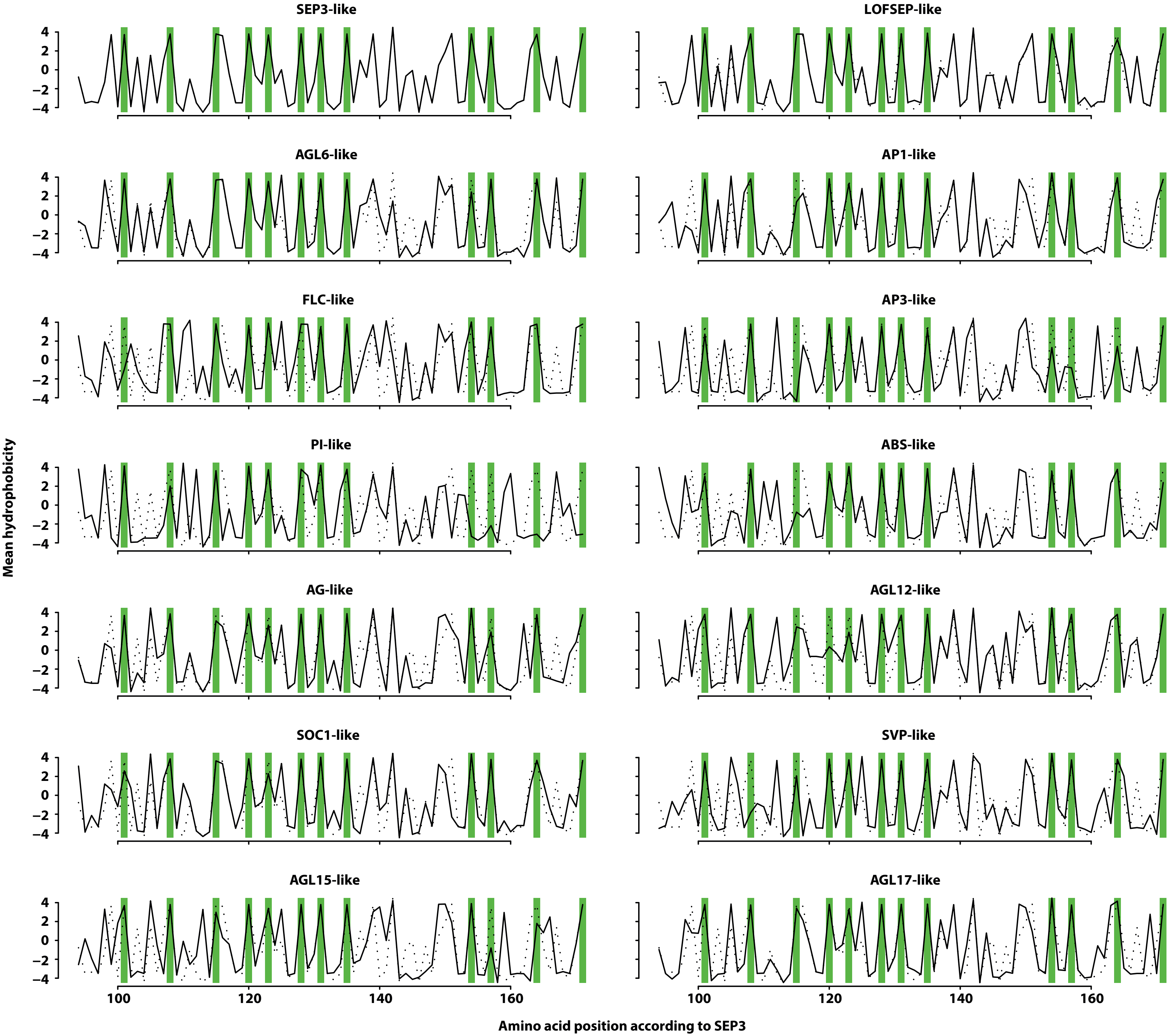

Supplementary Figure S7. Comparison of hydrophobicity pattern within the K-domain for all subfamilies of MIKC-type MADS-domain proteins. Each sub-chart depicts the mean hydrophobicity at each amino acid position within the K-domain of the respective subfamily. For better comparison the hydrophobicity values of SEP3-like proteins are shown as dotted lines in each sub-chart. Green bars highlight amino acid positions homologous to leucine residues that contribute to intra- and intermolecular interactions within a SEP3 homotetramer.

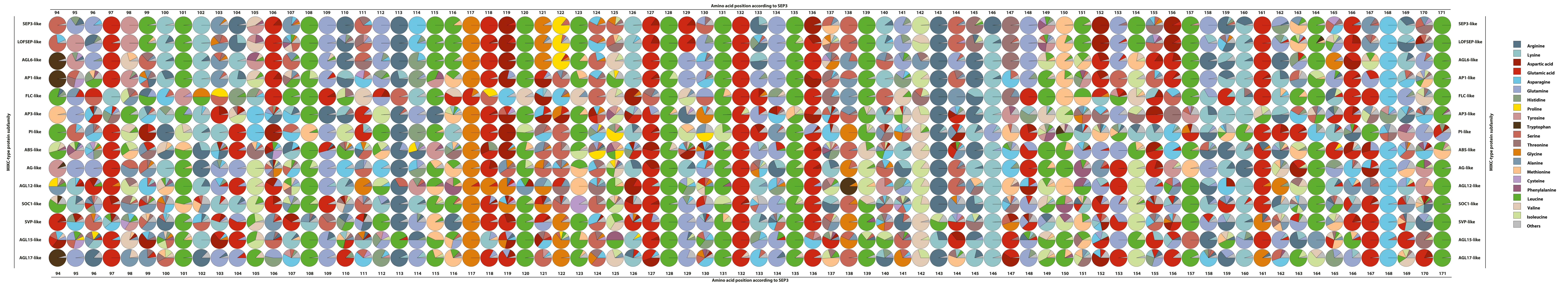

Supplementary Figure S8. Analysis of amino acid composition of the K-domain for all MIKC-type protein subfamilies. Each pie chart depicts the amino acid frequency at the corresponding position and for the respective subfamily. Amino acids that occurred in less than 5 % of the examined subset of sequences were condensed as 'others'. The vast majority of the positions shown vertically are homologous to each other. The only exception are position 151 to 171 of PI-like proteins. In this case, a gap was detected in the alignment but amino acids directly following the gap were included here.
